# A multivariate analysis with direct additive and inbreeding depression load effects

**DOI:** 10.1186/s12711-019-0521-3

**Published:** 2019-12-26

**Authors:** Luis Varona, Juan Altarriba, Carlos Moreno, María Martínez-Castillero, Joaquim Casellas

**Affiliations:** 1 Departamento de Anatomía Embriología y Genética Animal, Instituto Agroalimentario de Aragón (IA2), Universidad de Zaragoza, 50013 Saragossa, Spain; 2 Dipartimento di Agronomia Animali, Alimenti Risorce Naturali e Ambiente, Università degli Studi di Padova, 35122 Padua, Italy; 3 Departament de Ciència Animal i dels Aliments, Universitat Autònoma de Barcelona, 08193 Bellaterra, Spain

## Abstract

**Background:**

Inbreeding is caused by mating between related individuals and its most common consequence is inbreeding depression. Several studies have detected heterogeneity in inbreeding depression among founder individuals, and recently a procedure for predicting hidden inbreeding depression loads associated with founders and the Mendelian sampling of non-founders has been developed. The objectives of our study were to expand this model to predict the inbreeding loads for all individuals in the pedigree and to estimate the covariance between the inbreeding loads and the additive genetic effects for the trait of interest. We tested the proposed approach with simulated data and with two datasets of records on weaning weight from the Spanish Pirenaica and Rubia Gallega beef cattle breeds.

**Results:**

The posterior estimates of the variance components with the simulated datasets did not differ significantly from the simulation parameters. In addition, the correlation between the predicted and simulated inbreeding loads were always positive and ranged from 0.27 to 0.82. The beef cattle datasets comprised 35,126 and 75,194 records on weights between 170 and 250 days of age, and pedigrees of 308,836 and 384,434 individual-sire-dam entries for the Pirenaica and Rubia Gallega breeds, respectively. The posterior mean estimates of the variance of inbreeding depression loads were 29,967.8 and 28,222.4 for the Pirenaica and Rubia Gallega breeds, respectively. They were larger than those of the additive variance (695.0 and 439.8 for Pirenaica and Rubia Gallega, respectively), because they should be understood as the variance of the inbreeding depression achieved by a fully inbred (100%) descendant. Therefore, the inbreeding loads have to be rescaled for smaller inbreeding coefficients. In addition, a strong negative correlation (− 0.43 ± 0.10) between additive effects and inbreeding loads was detected in the Pirenaica, but not in the Rubia Gallega breed.

**Conclusions:**

The results of the simulation study confirmed the ability of the proposed procedure to predict inbreeding depression loads for all individuals in the populations. Furthermore, the results obtained from the two real datasets confirmed the variability in the inbreeding depression loads in both breeds and suggested a negative correlation of the inbreeding loads with the additive genetic effects in the Pirenaica breed.

## Background

Inbreeding is caused by mating between related individuals and is associated with changes in the mean and variance of quantitative traits [1]. Inbreeding depression, which results in a reduction in the phenotypic yield of fitness-related traits [2, 3], has been widely observed in animals, plants, and humans [2, 4] and is the most common consequence of inbreeding. The genetic basis of inbreeding depression stems from the high degree of homozygosity in inbred individuals, which reveals the presence of recessive alleles or losses in the advantage of over-dominance at heterozygous loci [2, 3].

Genetic variation within a population implies that inbreeding depression can vary depending on the genotype of the individuals whose alleles produce identity-by-descent (IBD) states in their progeny. Variability in inbreeding depression has been confirmed within ancestral lineages of Drosophila [5] and in sire families in dairy [6] and beef cattle [7]. Therefore, it is possible to define a specific hidden individual inbreeding depression load [8] (hereafter referred to as inbreeding load), which can be considered a hereditary trait [5, 9] with a phenotype that is only expressed when inbreeding occurs in its offspring.

Lacy et al. [10] proposed a founder decomposition of inbreeding, which assigns a different inbreeding depression to each founder of the population; yet, the common ancestors of inbred individuals are not restricted to the founding generation. Caballero and Toro [11] and García-Cortés et al. [12] proposed a Mendelian decomposition of the inbreeding coefficient that assigns partial inbreeding coefficients to the founders and to the Mendelian sampling of the non-founders. This decomposition was the basis for the development of a mixed-model approach that allows prediction of individual inbreeding loads [8]. However, this model assumes that the founding and non-founding effects on inbreeding depression are distributed independently, and cannot be used to predict the inbreeding load of individuals that do not have inbred progeny. In this study, we propose an alternative parameterization that can predict the inbreeding load for each individual in the pedigree, and can provide estimates of the covariance between the inbreeding loads and the additive genetic effects for the trait of interest. We tested the procedure with a simulation study and two large datasets of records on weaning weight in the Pirenaica and Rubia Gallega beef cattle breeds.

## Methods

### Theory

#### Inbreeding load

The recessive alleles that appear in homozygosity caused by IBD from a common ancestor in the paternal and maternal lineages can cause inbreeding depression. Each ancestor might have different sets of recessive alleles; thus, the genotype of each individual determines an inbreeding load that can be interpreted as the effects on the trait performance of its inbred descendants. Inbreeding load can be considered a heritable trait [5, 9] and, from the perspective of the ancestors, it acts additively because the alternative alleles never interact in their inbred progeny.

Thus, the polygenic inbreeding load ($i_{i}$) for the $i$th individual can be decomposed [8] as follows:

1 $$i_{i} = i_{s} + i_{d} + \varepsilon_{i} ,$$

where $i_{s}$ and $i_{d}$ are the inbreeding loads for its sire and dam, respectively, and $\varepsilon_{i}$ is its Mendelian sampling. Therefore, it follows that the distribution of the inbreeding loads is multivariate Gaussian as:

$${\mathbf{i}}\sim N\left( {0,{\mathbf{A}}\sigma_{i}^{2} } \right),$$

where ${\mathbf{A}}$ is the numerator relationship matrix [13], and $\upsigma_{i}^{2}$ is the additive genetic variance of the inbreeding loads.

#### Inbreeding decomposition

**Fig. 1:**
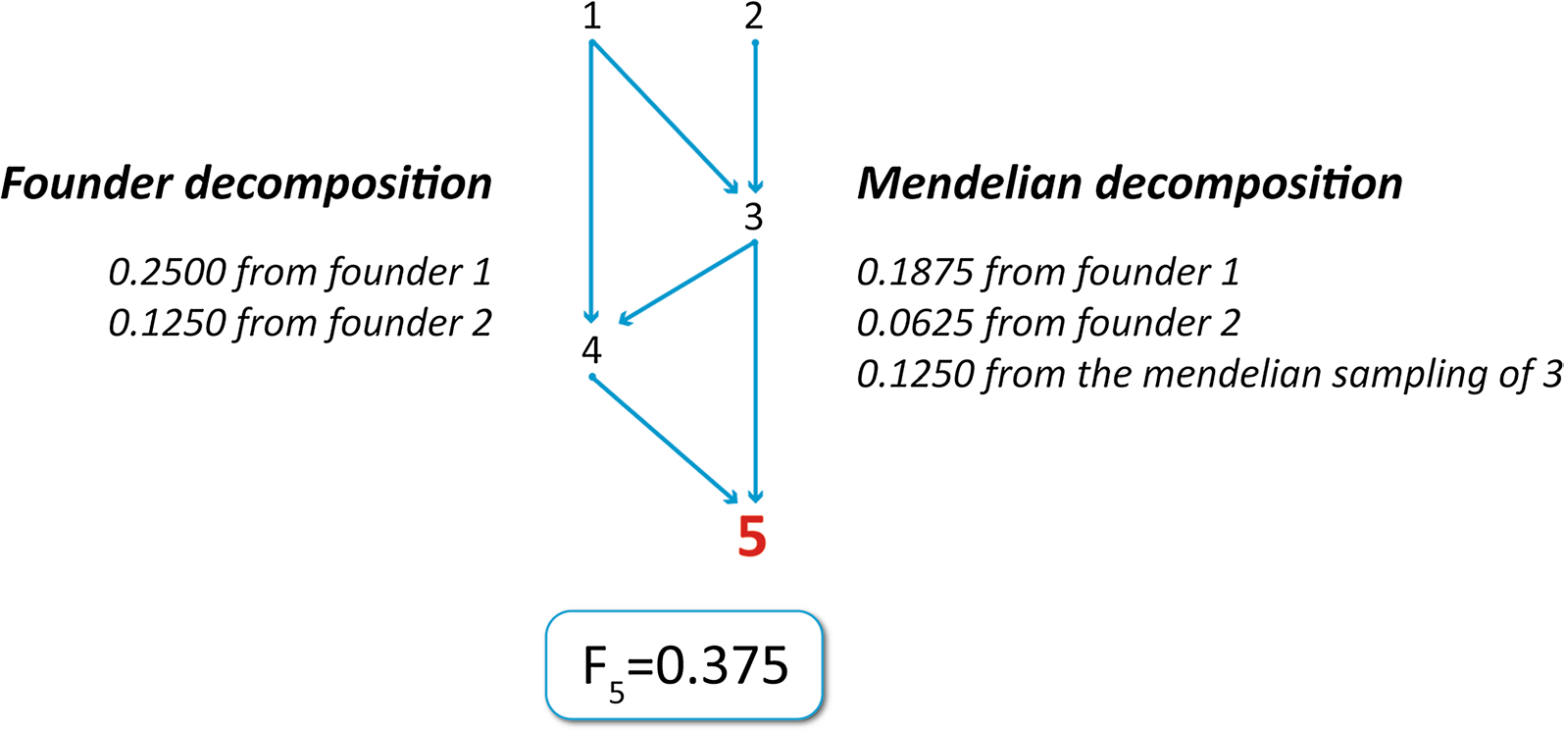
Founder and Mendelian decomposition of the inbreeding. Partial inbreeding coefficients for the fifth individual with the Founder and Mendelian decompositions of inbreeding

Traditionally, inbreeding is split into several units that are attributed to founding individuals [10] by tracing back the origin of the alleles that might be IBD in inbred individuals. Alternatively, inbreeding can be decomposed into the sources of the co-ancestry between the parents of each individual [11, 12], which might include the founders of the population and the Mendelian sampling of the non-founders. The difference between the two approaches is illustrated by a simple pedigree of five individuals (Fig. 1). Individual 5 has an inbreeding of 0.375. The founder decomposition splits it between two founders, and attributes 0.25 and 0.125 to individuals 1 and 2, respectively. In contrast, the Mendelian decomposition attributes 0.1875 and 0.0625 to founders 1 and 2, respectively, and 0.125 to the Mendelian sampling of non-founder 3. Note that, under the founder decomposition, the genealogical inbreeding is the sum of the partial coefficients from the founders, while under the Mendelian decomposition it is the sum of the partial coefficients from the founders and from the Mendelian sampling of the non-founders.

### Model

Based on the Mendelian decomposition of inbreeding, Casellas [8] proposed a linear model that explains the phenotypic data (${\mathbf{y}}$) with two vectors of random effects: the additive genetic (${\mathbf{a}}$), and one extra effect (${\varvec{\upvarepsilon}}_{{\mathbf{i}}}$) that is attributed to the inbreeding depression generated by the inbreeding loads of their ancestors (the founders and the Mendelian sampling of the non-founders). Thus,

2 $${\mathbf{y}} = {\mathbf{1}}^{\prime}\mu + {\mathbf{Za}} + {\varvec{\upvarepsilon}}_{{\mathbf{i}}} + {\mathbf{e}},$$

where $\mu$ is the general mean, ${\mathbf{e}}$ is the vector of the residuals, $1$ is a vector of ones with the same length of **y**, ${\mathbf{Z}}$ is the incidence matrix and ${\mathbf{T}}$ is the matrix that contains the partial inbreeding coefficients from the Mendelian decomposition and connects the phenotypic data of inbred individuals and the inbreeding loads of their common ancestors from the paternal and maternal lineages. Furthermore, ${\mathbf{a}}\sim N\left( {0,{\mathbf{A}}\upsigma_{\text{a}}^{2} } \right)$ and ${\mathbf{e}}\sim N\left( {0,{\mathbf{I}}\upsigma_{\text{e}}^{2} } \right)$, where ${\mathbf{A}}$ is the numerator relationship matrix and $\upsigma_{\text{a}}^{2}$ and $\upsigma_{\text{e}}^{2}$ are their associated variance components. Casellas [8] proposed that the distribution of ${\varvec{\upvarepsilon}}_{{\mathbf{i}}}$ is ${\varvec{\upvarepsilon}}_{{\mathbf{i}}} \sim N\left( {0,{\mathbf{I}}\upsigma_{\text{i}}^{2} } \right)$ with $\upsigma_{\text{i}}^{2}$ being the variance component of the inbreeding loads. However, this assumption is incorrect because the variance that is attributed to the inbreeding loads of the founders must be larger than the variance that is attributed to the Mendelian sampling of the non-founders. Thus, the appropriate distribution of $\upvarepsilon_{\text{i}}$ is $\upvarepsilon_{\text{i}} \sim N\left( {0,{\mathbf{Q}}\upsigma_{\text{i}}^{2} } \right)$, where ${\mathbf{Q}}$ is a diagonal matrix with a value 1 for the elements corresponding to founder individuals, and a value of $\frac{1}{2}\left( {1 - \frac{{F_{S} + F_{D} }}{2}} \right)$ for the elements corresponding to the non-founder individuals [11]. $F_{S}$ and $F_{D}$ are the genealogical inbreeding coefficient of the sire and dam of the individual, respectively.

In this study, we reparametrize the model in terms of individual inbreeding loads (${\mathbf{i}}$) that, given their additive nature, can be represented as follows:

$${\mathbf{i}} = {\mathbf{Pi}} + {\varvec{\upvarepsilon}}_{{\mathbf{i}}} ,$$

where ${\mathbf{P}}$ is a matrix with a diagonal of 0 s and 0.5 in the elements that link an individual with its sire and dam, such that it establishes a recurrent relationship between the inbreeding loads of each individual with their parents, as in Eq. (1). As a result, ${\varvec{\upvarepsilon}}_{{\mathbf{i}}} = \left( {{\mathbf{I}} - {\mathbf{P}}} \right){\mathbf{i}}$ and Model (2) can be rewritten as follows:

3 $${\mathbf{y}} = {\mathbf{1}}^{\prime}\mu + {\mathbf{Za}} + {\mathbf{Ki}} + {\mathbf{e}},$$

where ${\mathbf{K}} = {\mathbf{T}}\left( {{\mathbf{I}} - {\mathbf{P}}} \right)$ and ${\mathbf{i}}\sim N\left( {0,{\mathbf{A}}\upsigma_{\text{i}}^{2} } \right).$ As a result, a multiple trait model that includes the genetic covariance between additive effects and inbreeding loads can then be defined as:

4 $$\left( {\begin{array}{*{20}c} {\mathbf{a}} \\ {\mathbf{i}} \\ \end{array} } \right)\sim N\left( {\begin{array}{*{20}c} 0 \\ 0 \\ \end{array} ,{\mathbf{G}} \otimes {\mathbf{A}}} \right),$$

where $${\mathbf{G}} = \left( {\begin{array}{*{20}c} {\upsigma_{\text{a}}^{2} } & {\upsigma_{\text{ai}} } \\ {\upsigma_{\text{ai}} } & {\upsigma_{\text{i}}^{2} } \\ \end{array} } \right),$$ and $\upsigma_{\text{ai}}$ is the covariance between additive effects and inbreeding loads.

### Example

Given the pedigree of five individuals (Fig. 1), we assume that individuals 3, 4, and 5 have phenotypes 113, 87, and 96, respectively. Applying the method proposed by García-Cortés et al. [12] a partial inbreeding coefficient of 0.250 is obtained for the animal 4 generated by individual 1, and three partial inbreeding coefficients (0.1875, 0.0625, and 0.125) for animal 5, generated by individuals 1, 2, and 3, respectively. Therefore, the mixed-model equations for the implementation of the model defined by Eq. (3) requires the following vector and matrices:

$${\mathbf{y}} = \left[ {\begin{array}{*{20}c} {113} \\ {87} \\ {96} \\ \end{array} } \right],$$

$${\mathbf{Z}} = \left[ {\begin{array}{*{20}c} 0 &\quad 0 &\quad 1 &\quad 0 &\quad 0 \\ 0 &\quad 0 &\quad 0 &\quad 1 &\quad 0 \\ 0 &\quad 0 &\quad 0 &\quad 0 &\quad 1 \\ \end{array} } \right],$$

$${\mathbf{K}} = {\mathbf{T}}\left( {{\mathbf{I}} - {\mathbf{P}}} \right) = \left[ {\begin{array}{*{20}c} 0 &\quad 0 &\quad 0 &\quad 0 &\quad 0 \\ {0.25} &\quad 0 &\quad 0 &\quad 0 &\quad 0 \\ {0.125} &\quad 0 &\quad {0.125} &\quad 0 &\quad 0 \\ \end{array} } \right],$$

$${\text{where}}\;{\mathbf{T}} = \left[ {\begin{array}{*{20}c} 0 &\quad 0 &\quad 0 &\quad 0 &\quad 0 \\ {0.25} &\quad 0 &\quad 0 &\quad 0 &\quad 0 \\ {0.1875} &\quad {0.0625} &\quad {0.125} &\quad 0 &\quad 0 \\ \end{array} } \right],$$

$${\text{and}}\;{\mathbf{I}} - {\mathbf{P}} = \left[ {\begin{array}{*{20}c} 1 &\quad 0 &\quad 0 &\quad 0 &\quad 0 \\ 0 &\quad 1 &\quad 0 &\quad 0 &\quad 0 \\ { - 0.5} &\quad { - 0.5} &\quad 1 &\quad 0 &\quad 0 \\ { - 0.5} &\quad 0 &\quad { - 0.5} &\quad 1 &\quad 0 \\ 0 &\quad 0 &\quad { - 0.5} &\quad { - 0.5} &\quad 1 \\ \end{array} } \right].$$

In addition, the numerator relationship matrix (${\mathbf{A}}$) and its inverse (${\mathbf{A}}^{ - 1}$) are as follows:

$${\mathbf{A}} = \left[ {\begin{array}{*{20}c} 1 &\quad 0 &\quad {0.500} &\quad {0.750} &\quad {0.625} \\ 0 &\quad 1 &\quad {0.500} &\quad {0.250} &\quad {0.375} \\ {0.500} &\quad {0.500} &\quad 1 &\quad {0.750} &\quad {0.875} \\ {0.750} &\quad {0.250} &\quad {0.750} &\quad {1.250} &\quad 1 \\ {0.625} &\quad {0.375} &\quad {0.875} &\quad 1 &\quad {1.375} \\ \end{array} } \right],$$

and

$${\mathbf{A}}^{ - 1} = \left[ {\begin{array}{*{20}c} 2 &\quad {0.5} &\quad { - 0.5} &\quad { - 1} &\quad 0 \\ {0.5} &\quad {1.5} &\quad { - 1} &\quad 0 &\quad 0 \\ { - 0.5} &\quad { - 1} &\quad {3.071} &\quad { - 0.429} &\quad { - 1.143} \\ { - 1} &\quad 0 &\quad { - 0.429} &\quad {2.571} &\quad { - 1.143} \\ 0 &\quad 0 &\quad { - 1.143} &\quad { - 1.143} &\quad {2.286} \\ \end{array} } \right],$$

which leads to the following mixed-model equations:

$$\begin{aligned}& \left[ {\begin{array}{*{20}c} {1^{\prime}1} &\quad {1^{\prime}{\mathbf{Z}}} &\quad {1^{\prime}{\mathbf{K}}} \\ {{\mathbf{Z^{\prime}}}1} &\quad {{\mathbf{Z^{\prime}Z}} + {\mathbf{A}}^{ - 1} {\text{g}}^{11}\upsigma_{\text{e}}^{2} } &\quad {{\mathbf{Z^{\prime}K}} + {\mathbf{A}}^{ - 1} {\text{g}}^{12}\upsigma_{e}^{2} } \\ {{\mathbf{K^{\prime}}}1} &\quad {{\mathbf{K^{\prime}Z}} + {\mathbf{A}}^{ - 1} {\text{g}}^{21}\upsigma_{\text{e}}^{2} } &\quad {{\mathbf{K^{\prime}K}} + {\mathbf{A}}^{ - 1} {\text{g}}^{22}\upsigma_{e}^{2} } \\ \end{array} } \right]\left[ {\begin{array}{*{20}c} {\hat{\mu }} \\ {{\hat{\mathbf{a}}}} \\ {{\hat{\mathbf{i}}}} \\ \end{array} } \right] \\ &\quad= \left[ {\begin{array}{*{20}c} {1^{\prime}{\mathbf{y}}} \\ {{\mathbf{Z^{\prime}y}}} \\ {{\mathbf{K^{\prime}y}}} \\ \end{array} } \right], \end{aligned}$$

where ${\text{g}}^{11}$, ${\text{g}}^{12}$, ${\text{g}}^{22}$ are the elements of the inverse (${\mathbf{G}}^{ - 1}$) of the covariance matrix between the additive and the inbreeding load effects. Assuming $\upsigma_{\text{e}}^{2} = 10$, $\upsigma_{\text{a}}^{2} = 2$, $\upsigma_{\text{i}}^{2} = 1$, and $\upsigma_{\text{ai}} = - 0.25$, then

$${\mathbf{G}}^{ - 1} = \left[ {\begin{array}{*{20}c} {{\text{g}}^{11} } & {{\text{g}}^{12} } \\ {{\text{g}}^{21} } & {{\text{g}}^{22} } \\ \end{array} } \right] = \left[ {\begin{array}{*{20}c} 2 & { - 0.25} \\ { - 0.25} & 1 \\ \end{array} } \right]^{ - 1} = \left[ {\begin{array}{*{20}c} {0.516} & {0.129} \\ {0.129} & {1.032} \\ \end{array} } \right],$$

and the equations become the following:

$$\left[ {\begin{array}{*{20}c} 3 &\quad 0 &\quad 0 &\quad 1 &\quad 1 &\quad 1 &\quad {0.375} &\quad 0 &\quad {0.125} &\quad 0 &\quad 0 \\ 0 &\quad {1.032} &\quad {0.258} &\quad { - 0.258} &\quad { - 0.516} &\quad 0 &\quad {0.258} &\quad {0.0645} &\quad { - 0.0645} &\quad { - 0.129} &\quad 0 \\ 0 &\quad {0.258} &\quad {0.774} &\quad { - 0.516} &\quad 0 &\quad 0 &\quad {0.0645} &\quad {0.1935} &\quad { - 0.129} &\quad 0 &\quad 0 \\ 1 &\quad { - 0.258} &\quad { - 0.516} &\quad {2.5849} &\quad { - 0.2211} &\quad { - 0.5897} &\quad { - 0.0645} &\quad { - 0.129} &\quad {0.3962} &\quad { - 0.0553} &\quad { - 0.1474} \\ 1 &\quad { - 0.516} &\quad 0 &\quad { - 0.2211} &\quad {2.327} &\quad { - 0.5897} &\quad {0.121} &\quad 0 &\quad { - 0.0553} &\quad {0.332} &\quad { - 0.1474} \\ 1 &\quad 0 &\quad 0 &\quad { - 0.5897} &\quad { - 0.5897} &\quad {2.1794} &\quad {0.125} &\quad 0 &\quad { - 0.022} &\quad { - 0.1474} &\quad {0.2949} \\ {0.375} &\quad {0.258} &\quad {0.0645} &\quad { - 0.0645} &\quad {0.121} &\quad {0.125} &\quad {2.142} &\quad {0.516} &\quad { - 0.500} &\quad { - 1.032} &\quad 0 \\ 0 &\quad {0.0645} &\quad {0.1935} &\quad { - 0.129} &\quad 0 &\quad 0 &\quad { - 0.500} &\quad {1.548} &\quad { - 1.032} &\quad 0 &\quad 0 \\ {0.125} &\quad { - 0.0645} &\quad { - 0.129} &\quad {0.3962} &\quad { - 0.0552} &\quad { - 0.022} &\quad {1.0465} &\quad { - 1.032} &\quad {3.185} &\quad { - 0.4423} &\quad { - 1.1794} \\ 0 &\quad { - 0.129} &\quad 0 &\quad { - 0.0553} &\quad {0.3317} &\quad { - 0.1474} &\quad { - 1.032} &\quad 0 &\quad { - 0.4423} &\quad {2.6537} &\quad { - 1.1794} \\ 0 &\quad 0 &\quad 0 &\quad { - 0.1474} &\quad { - 0.1474} &\quad {0.2948} &\quad 0 &\quad 0 &\quad { - 1.1794} &\quad { - 1.1794} &\quad {2.3589} \\ \end{array} } \right]\left[ {\begin{array}{*{20}c} {\hat{\mu }} \\ {{\hat{\mathbf{a}}}_{1} } \\ {{\hat{\mathbf{a}}}_{2} } \\ \begin{aligned} {\hat{\mathbf{a}}}_{3} \hfill \\ {\hat{\mathbf{a}}}_{4} \hfill \\ {\hat{\mathbf{a}}}_{5} \hfill \\ {\hat{\mathbf{i}}}_{1} \hfill \\ {\hat{\mathbf{i}}}_{2} \hfill \\ {\hat{\mathbf{i}}}_{3} \hfill \\ {\hat{\mathbf{i}}}_{4} \hfill \\ {\hat{\mathbf{i}}}_{5} \hfill \\ \end{aligned} \\ \end{array} } \right] = \left[ {\begin{array}{*{20}c} {296} \\ 0 \\ 0 \\ {113} \\ {87} \\ {96} \\ {33.75} \\ 0 \\ {12} \\ 0 \\ 0 \\ \end{array} } \right],$$

with solutions:

$$ \left[ {\begin{array}{*{20}c} {\hat{\mu }} \\ {{\hat{\mathbf{a}}}_{1} } \\ {{\hat{\mathbf{a}}}_{2} } \\ {{\hat{\mathbf{a}}}_{3} } \\ {{\hat{\mathbf{a}}}_{4} } \\ {{\hat{\mathbf{a}}}_{5} } \\ {{\hat{\mathbf{i}}}_{1} } \\ {{\hat{\mathbf{i}}}_{2} } \\ {{\hat{\mathbf{i}}}_{3} } \\ {{\hat{\mathbf{i}}}_{4} } \\ {{\hat{\mathbf{i}}}_{5} } \\ \end{array} } \right] = \left[ {\begin{array}{*{20}c} {104.88} \\ { - 2.64} \\ {4.37} \\ {1.96} \\ { - 9.81} \\ { - 6.24} \\ { - 4.50} \\ { - 13.49} \\ { - 9.37} \\ { - 5.41} \\ { - 7.07} \\ \end{array} } \right]. $$

### Simulation

A pedigree of 30,000 individuals arranged in six discrete generations of 5000 individuals (2500 sires and 2500 dams) was simulated. Each generation was obtained from the random mating of 20 sires and 500 dams that were chosen randomly from the previous generation. Thus, neither selection nor purging were simulated. Once the pedigree was obtained, the direct additive genetic effects (${\mathbf{a}}$) and the inbreeding loads (${\mathbf{i}}$) were simulated based on the multivariate distribution from Eq. (4). The vector of the phenotypic records (${\mathbf{y}}$) was generated by Eq. (3). The partial inbreeding coefficients required for the ${\mathbf{T}}$ matrix were derived from the procedure of García-Cortés et al. [12].

Two cases (i) and (ii) were simulated based on the following parameters:

i. $$\mu$$ = 100, $$\upsigma_{\text{a}}^{2}$$ = 100, $$\upsigma_{\text{i}}^{2}$$ = 10,000, $$\upsigma_{\text{ai}}$$ = − 500, r(**a**, **i**) = − 0.5 and $$\upsigma_{\text{e}}^{2}$$ = 100,
ii. $$\mu$$ = 100, $$\upsigma_{\text{a}}^{2}$$ = 100, $$\upsigma_{\text{i}}^{2}$$ = 1000, $$\upsigma_{\text{ai}}$$ = 0, r(**a**, **i**) = 0.0 and $$\upsigma_{\text{e}}^{2}$$ = 100,
  $${\text{where}}\;{\text{r}}\left( {{\mathbf{a}},{\mathbf{i}}} \right) = \frac{{\upsigma_{\text{ai}} }}{{\upsigma_{\text{a}}\upsigma_{\text{i}} }}.$$

The simulated variances of inbreeding loads $\left( {\upsigma_{\text{i}}^{2} } \right)$ were greater than the additive variances $\left( {\upsigma_{\text{a}}^{2} } \right)$ as they reflect the inbreeding depression achieved by a completely inbred (100%) descendent. The percentage of phenotypic variance explained by the inbreeding depression loads depends on the magnitude of the partial inbreeding coefficients. Therefore, in the first simulation case (i), the variance of inbreeding load for a partial inbreeding coefficient of 0.10 will be equal to the additive variance (100), whereas in the second (ii), it will be a tenth part (10).

The simulated datasets were analyzed under Model (3). We used a Bayesian approach which included uniform priors for the mean ($\mu$) and for the variance components (${\mathbf{G}}$) and $\upsigma_{\text{e}}^{2}$, and a multivariate Gaussian distribution for ${\mathbf{a}}$ and ${\mathbf{i}}$. The analysis was implemented through a Gibbs Sampler [14] with two chains of 550,000 iterations, after discarding the first 50,000 iterations.

### Beef cattle data

The dataset included 35,126 (Pirenaica) and 75,194 (Rubia Gallega) records on live weights (one per individual) between 170 and 250 days of age, which were provided by the two breeding associations and had been recorded since 1989 and 1991, respectively, until 2018. The average (± standard deviation) weights in the Pirenaica and the Rubia Gallega breeds were 258.4 kg (± 58.0 kg) and 281.4 kg (± 46.7 kg), respectively. The genealogical information dated back to 1920 (Pirenaica) and 1955 (Rubia Gallega), and comprised 308,836 and 384,434 individual-sire-dam records, respectively. The average depth of the pedigree of the individuals for which there was phenotypic information was 5.9 and 3.9 generations for the Pirenaica and the Rubia Gallega breeds, respectively. In the Pirenaica breed, 248,471 (80.5%) individuals were inbred and had an average inbreeding (± standard deviation) coefficient of 0.030 (± 0.056). In the Rubia Gallega breed, inbreeding affected 176,048 (45.8%) individuals, and the average inbreeding coefficient (± standard deviation) was 0.020 (± 0.048). The evolutions of the average inbreeding and the percentage of inbred individuals from 1975 to 2017 are shown in Additional file 1: Figures S1 and Additional file 2: Figure S2 for Pirenaica and Rubia Gallega, respectively.

The partial inbreeding coefficients from the Mendelian decomposition of inbreeding were derived from the procedure of García-Cortés et al. [12], which generated 16,099,374 and 5,080,457 coefficients from 8721 and 3601 ancestors in the Pirenaica and Rubia Gallega breeds, respectively. The average partial inbreeding coefficients (± standard deviation) were 5.7 $\times$ 10^−4^ (± 4.2 $\times$ 10^−3^) in the Pirenaica and 1.5 $\times$ 10^−3^ (± 6.9 $\times$ 10^−3^) in the Rubia Gallega breed. Most of the partial inbreeding coefficients were very low (Table 1); 99.3 and 93.0%, and 97.4 and 81.2% were lower than 0.01 and 0.001 in the Pirenaica and Rubia Gallega breeds, respectively.

**Table 1:** Distribution of partial inbreeding coefficients (number and percentage) based on their magnitude in the Pirenaica and Rubia Gallega breeds

Partial inbreeding	Pirenaica	Rubia Gallega
< 10-4	10,996,924 (68.30%)	1,564,740 (30.80%)
10-4-10-3	3,967,842 (24.65%)	2,559,733 (50.38%)
10-3-10-2	1,028,578 (6.39%)	823,410 (16.21%)
10-1-10-2	126,238 (0.78%)	124,155 (2.44%)
> 10-1	9792 (0.06%)	8.419 (0.17%)

Once the partial inbreeding coefficients from the Mendelian decomposition of inbreeding were available, the phenotypic data were analyzed with the following model:

$${\mathbf{y}} = {\mathbf{t}}c + {\mathbf{Xb}} + {\mathbf{Wp}} + {\mathbf{Za}} + {\mathbf{Ki}} + {\mathbf{e}},$$

where ${\mathbf{t}}$ is the vector of age at recording, ${\mathbf{b}}$ is the vector of systematic effects, which included sex and age of the dam (15 levels), ${\mathbf{p}}$ is the vector of herd-year-season effects (Pirenaica, 6503 levels and Rubia Gallega, 5251 levels), and ${\mathbf{a}}$, ${\mathbf{i}}$, and ${\mathbf{e}}$ are the vectors of direct genetic, inbreeding load, and residual effects, respectively. Furthermore, $c$ is the covariate with age at recording, and ${\mathbf{X}}$, ${\mathbf{W}}$, ${\mathbf{Z}}$, and ${\mathbf{K}}$ are the appropriate incidence matrices.

Under a hierarchical Bayesian analysis, it was assumed that prior distributions for the permanent environmental, additive, and inbreeding load effects followed a multivariate Gaussian distribution:

$${\mathbf{p}}\sim {\text{N}}\left( {0,{\mathbf{I}}\sigma_{\text{p}}^{2} } \right),$$

where $\upsigma_{\text{p}}^{2}$ is the variance of the permanent environmental effects and

$$\left( {\begin{array}{*{20}c} {\mathbf{u}} \\ {\mathbf{i}} \\ \end{array} } \right)\sim N\left( {\begin{array}{*{20}c} 0 \\ 0 \\ \end{array} ,{\mathbf{G}} \otimes {\mathbf{A}}} \right),$$

as in Model (4).

The prior distribution for systematic effects were uniform between − M and M, being M a very large value. The prior distributions for $\upsigma_{\text{p}}^{2}$ was an inverted Chi-square $\left( {\chi^{ - 2} \left( {s,v} \right)} \right)$ distribution with parameters $s = 0$ and $v = - 2$, and the prior distribution for ${\mathbf{G}}$ were inverted Chi-square and inverted Wishart $\left( {IW\left( {{\mathbf{S}},v} \right)} \right)$ distribution with:

$${\mathbf{S}} = \left( {\begin{array}{*{20}c} 0 & 0 \\ 0 & 0 \\ \end{array} } \right),$$

and $v = - 3$, that reduces to a uniform distribution. The Gibbs sampler [14] was implemented with two chains of 550,000 iterations, after discarding the first 50,000 iterations. Convergence was confirmed by visual inspection of the chains (see Additional file 3: Figures S3 and Additional file 4: Figure S4) and by the use of the CODA package in the R software [15].

## Results

### Simulation study

**Fig. 2:**
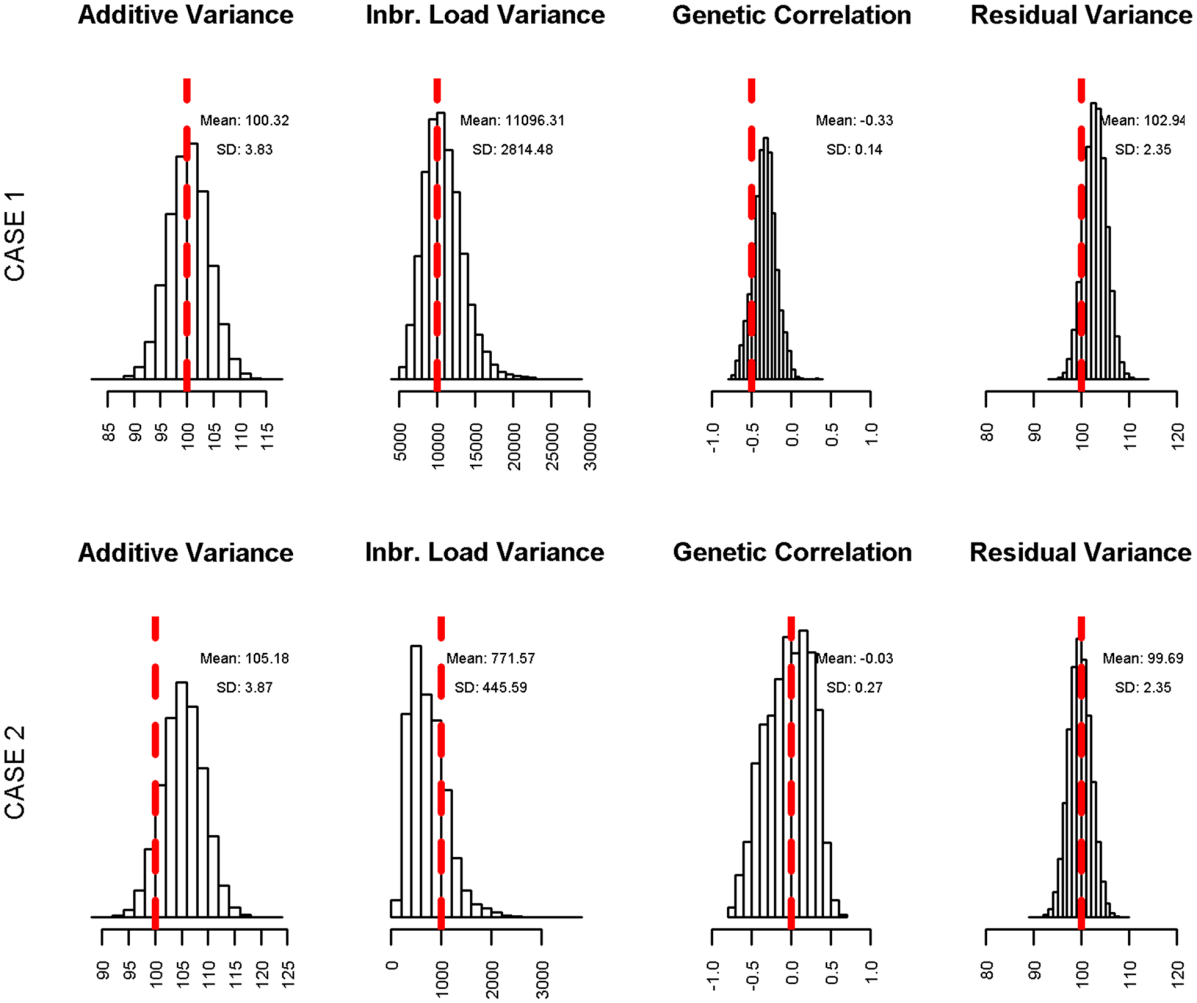
Posterior distributions of the variance components in the simulation study. Posterior distributions of the additive variance $\upsigma_{\text{a}}^{2}$, inbreeding load variance $\upsigma_{\text{i}}^{2}$, genetic correlation ${\text{r}}\left( {{\mathbf{a}},{\mathbf{i}}} \right)$ and residual variance $\upsigma_{\text{e}}^{2}$ from the two simulation cases with the values used in the simulation (vertical red line)

**Fig. 3:**
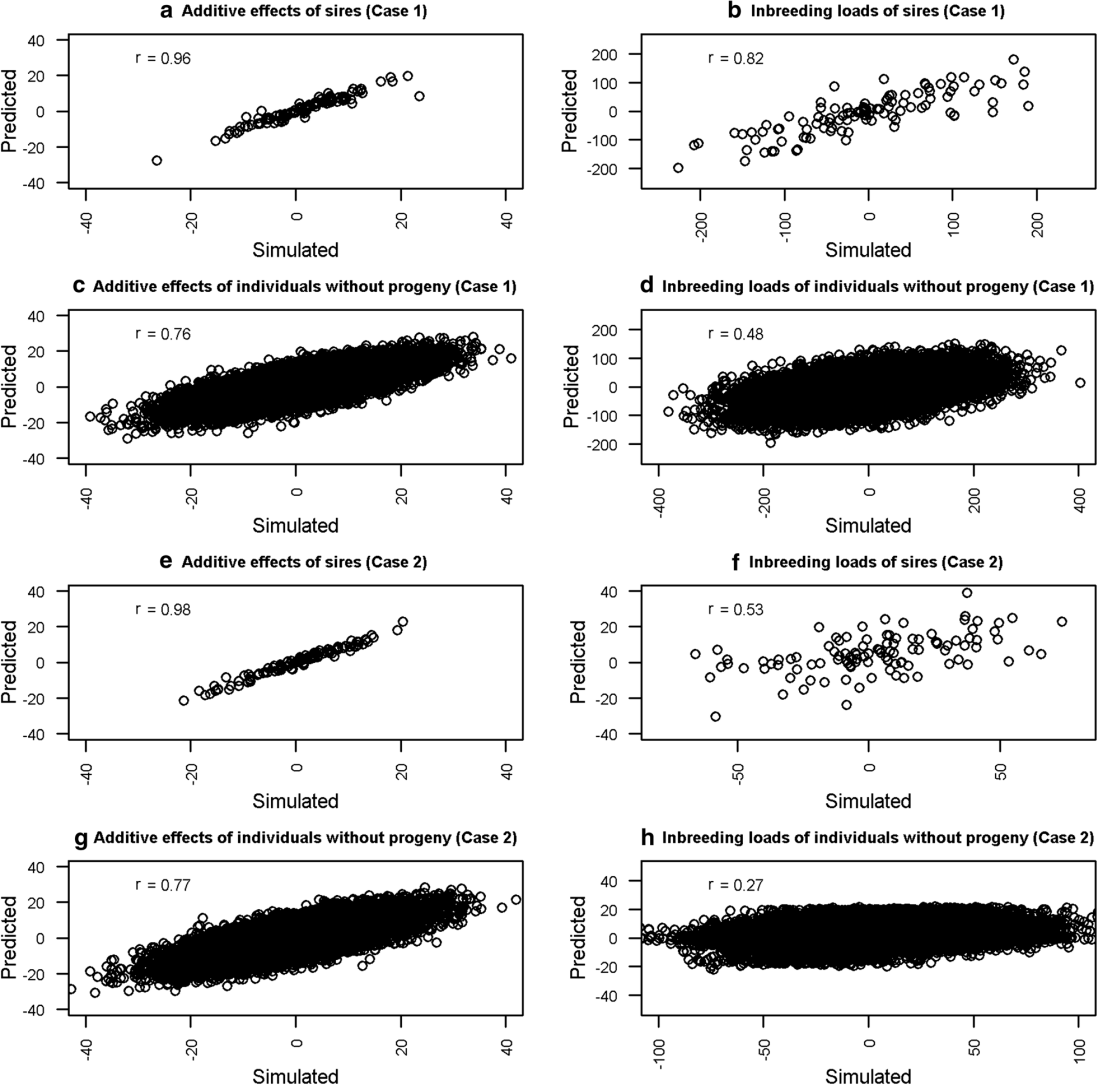
Relationship between simulated and predicted additive and inbreeding load effects in the simulation study. Simulated additive and inbreeding load effects (x axis) and their predictions (y axis) and correlation coefficients between them (r) for sires (**a**, **b**, **e** and **f**) and individuals without progeny (**c**, **d**, **g** and **h**). **a**–**d** Correspond to the first case of simulation and **e**–**h** to the second case

The posterior distributions of $\upsigma_{\text{a}}^{2}$, $\upsigma_{\text{i}}^{2}$, ${\text{r}}\left( {{\mathbf{a}},{\mathbf{i}}} \right)$ and $\upsigma_{\text{e}}^{2}$ obtained from the simulation study are shown in Fig. 2. The variance components used in the simulation were within the posterior mean ± 2 times the posterior standard deviation. Figure 3 shows the plots of the simulated direct genetic and inbreeding load effects and their predictions (posterior mean estimates) for sires and for individuals without progeny. In addition, the correlations between the simulated and predicted effects for sires and individuals without progeny are shown. The correlations between the direct genetic effects and their predictions ranged from 0.76 (individuals without progeny in case (i)) to 0.98 (sires in case (ii)), and were always higher than their counterparts for the inbreeding loads. Nevertheless, the correlations between simulated and predicted inbreeding loads were always positive, and ranged from 0.27 (individuals without progeny in case (ii)) to 0.82 (sires in case (i)).

### Beef cattle datasets

The posterior mean estimates of the variance components in the Pirenaica and Rubia Gallega breeds are in Table 2. The posterior mean estimates of the additive genetic variance and inbreeding load variance components were equal to 695.0 kg^2^ and 29,966.8 kg^2^ in Pirenaica, and 439.8 kg^2^ and 28,222.4 kg^2^ in Rubia Gallega, respectively. The posterior mean estimates of the genetic correlation between additive genetic and inbreeding load effects were − 0.43 in Pirenaica and − 0.04 in Rubia Gallega. The posterior mean estimates of the permanent environmental and residual variances were 1035.2 kg^2^ and 483.1 kg^2^ in Pirenaica, and 320.0 kg^2^ and 1018.2 kg^2^ in Rubia Gallega.

**Table 2:** Posterior mean estimates (and posterior standard deviation) of variance components in the Pirenaica and Rubia Gallega breeds

	Population	
	Pirenaica	Rubia Gallega
$\upsigma_{\text{a}}^{2}$	695.016 (25.688)	439.803 (18.121)
$\upsigma_{\text{i}}^{2}$	29,966.800 (5868.275)	28,222.360 (5454.273)
${\text{r}}\left( {{\mathbf{a}},{\mathbf{i}}} \right)$	− 0.429 (0.102)	− 0.043 (0.087)
$\upsigma_{\text{p}}^{2}$	1035.209 (27.714)	320.023 (11.273)
$\upsigma_{\text{e}}^{2}$	483.060 (15.010)	1018.207 (12.612)

$\upsigma_{\text{a}}^{2}$ is the additive genetic variance, $\upsigma_{\text{i}}^{2}$ is the variance of individual inbreeding loads, ${\text{r}}\left( {{\mathbf{a}},{\mathbf{i}}} \right)$ is the correlation between additive genetic and individual inbreeding loads, $\upsigma_{\text{p}}^{2}$ is the permanent environmental variance and $\upsigma_{\text{e}}^{2}$ is the residual variance

**Fig. 4:**
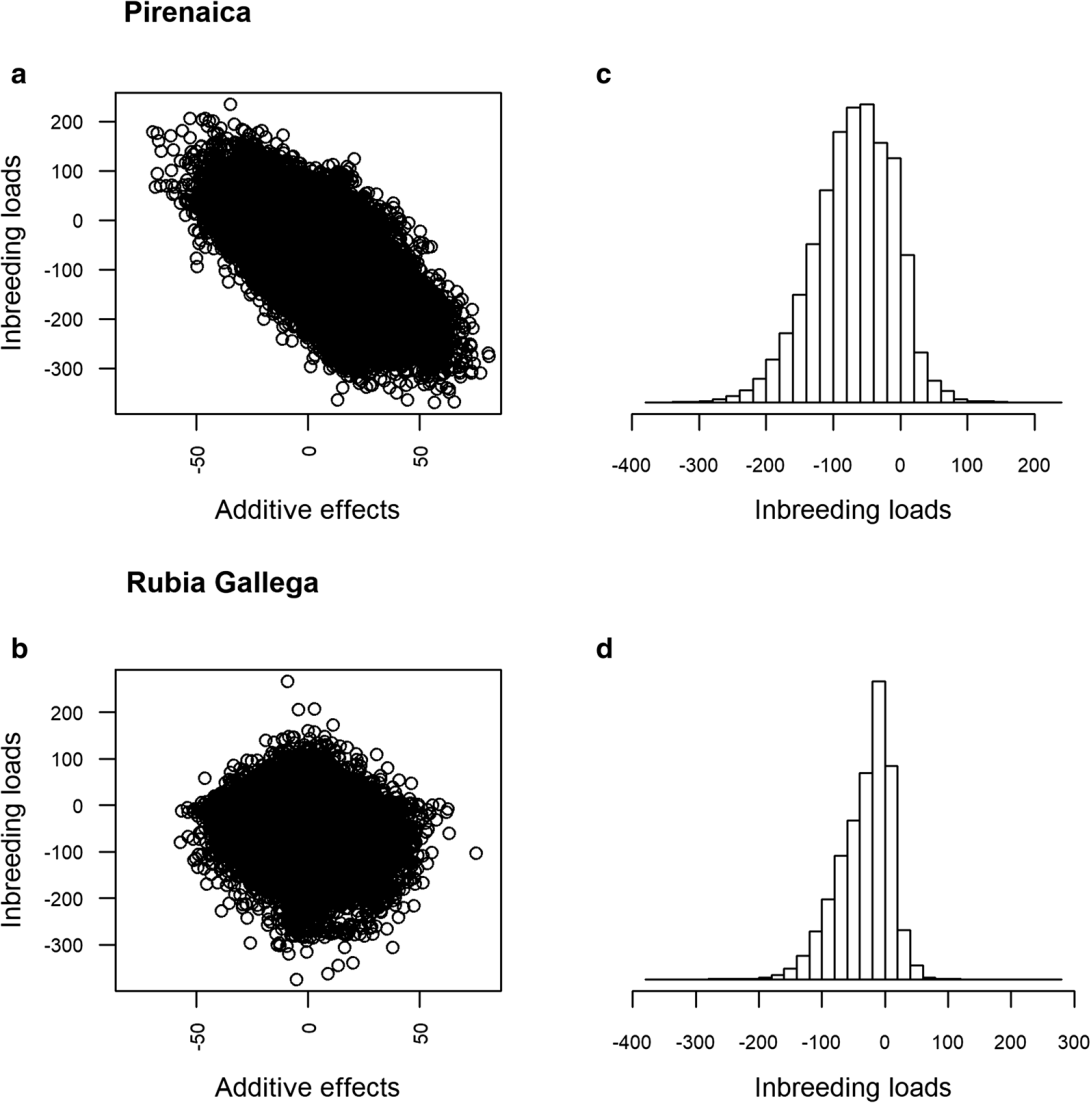
Relationship between predicted additive and inbreeding load effects and distribution of inbreeding load effects for weaning weight in Pirenaica and Rubia Gallega. Predictions of the additive (x axis) and inbreeding load (y axis) effects for weaning weight in Pirenaica (**a**) and Rubia Gallega (**b**) and histograms of predictions of inbreeding load effects for weaning weight in Pirenaica (**c**) and Rubia Gallega (**d**)

Figure 4 presents, for each breed, a bivariate plot of the predictions (posterior mean estimates) of the direct additive and inbreeding load effects and a histogram that represents the distribution of the predicted inbreeding loads. In the Pirenaica breed, the bivariate plot indicated a clear negative relationship between direct genetic and inbreeding load effects, which was not apparent in the Rubia Gallega breed. The average (and standard deviation) of the predicted inbreeding loads were -69.3 (58.6) kg and − 32.3 (41.7) kg in Pirenaica and Rubia Gallega breeds, respectively, and ranged from − 369.1 to 234.7 in Pirenaica and from − 374.1 to 266.1 in Rubia Gallega. The proportion of individuals in the Pirenaica and Rubia Gallega breeds that had a positive predicted inbreeding load was equal to 10.5% and 22.5%, respectively.

## Discussion

Inbreeding load and inbreeding depression are heterogeneous among populations [5] or families [6, 7], as inbreeding depression is affected by the variants of recessive alleles in each population, or even in each ancestor that generates inbreeding. Variability in inbreeding depression has been confirmed in several livestock populations [16–21] with the approach proposed by Lacy et al. [10], which is consistent with the results of previous studies [22–24] that revealed heterogeneity in the contribution of genomic regions to inbreeding depression in several traits and species.

Management of inbreeding to prevent the undesirable consequences is an important aspect of conservation genetics [25] and in livestock and plant breeding programs [26, 27]. Purging is a natural process of selection against recessive alleles that are expressed in the homozygous state [28]. Purging during past generations can be inferred in pedigreed populations [29–31], and evidence of purging has been detected in natural [32–34] and livestock [29, 35] populations.

Genetic variability of the inbreeding loads among individuals within a population can be taken into account in ‘artificial’ purging strategies, to avoid the use of those individuals with poor inbreeding loads for reproductive purposes. However, it requires prediction of the inbreeding loads of young individuals without inbred progeny. The approach proposed by Lacy et al. [10] is useful for describing the heterogeneity of inbreeding depression among founders, yet in most livestock populations, the founders live several generations before the current candidates for selection and, therefore, the prediction of the inbreeding loads based on Lacy’s approach is not useful for practical ‘artificial’ purging strategies. Casellas [8] proposed an approach that uses the Mendelian decomposition of inbreeding and provides prediction of the inbreeding loads from both founders and the Mendelian sampling of the non-founders. However, it can only provide prediction of the inbreeding loads of the individuals that contribute to the inbreeding of individuals with known phenotypes. Moreover, its estimates of the variance of the inbreeding loads are biased, since it ignores the reduction in variance due to the Mendelian segregation of the progeny.

Although the approach proposed by Casellas [8] was the basis of the approach proposed in our study, rather than using directly the partial inbreeding coefficients derived from García-Cortes et al. [12], the coefficients were transformed by the I–P recursive transformation in order to predict the total individual inbreeding load (${\mathbf{i}}$). The parametrization is crucial because it allows the assumption that individual inbreeding loads follow a multivariate Gaussian distribution and permits the use of the numerator relationship matrix (${\mathbf{A}}$) to predict the inbreeding loads of individuals that have yet to contribute to inbreeding but have relatives that have done so. The approach is illustrated with the results obtained from a simulation study, which used the same model of analysis to generate the simulated datasets. In spite of the limited amount of data, the posterior distribution of the variance components did not differ significantly from the simulated ones (Fig. 2), which suggests that there is enough information to recover the variance of inbreeding loads and their covariance with the direct additive effects. The most outstanding result of the simulation study is that it demonstrates the ability of prediction of the inbreeding loads of individuals without progeny, since the correlations between the predicted and simulated inbreeding loads were 0.27 and 0.48 in the first (i) and second (ii) case of simulation, respectively (Fig. 3). The higher correlation in the second case was caused by the larger $\upsigma_{\text{i}}^{2}$ used in the simulation. The correlations obtained from the individuals without progeny were lower than those for the sires, which had many inbred progeny, and they were also lower than the correlations between simulated and predicted additive effects. Nonetheless, these results might provide a basis for the development of “artificial” purging strategies in order to avoid the undesirable consequences of inbreeding depression. One approach might be to develop a selection index by weighting the breeding values and inbreeding loads in an appropriate way.

Implementation of our model involved the construction of the mixed-model equations, which followed the same rationale as that of the maternal animal model [36]. The model attributes two genetic effects to each individual; one, i.e. ${\mathbf{a}}$ that it is expressed directly in the individual phenotype, and another, i.e. ${\mathbf{i}}$ that it is expressed only in the phenotype of its inbred descendants. In spite of the complexity of the model, the results obtained from the simulation study (Fig. 2) demonstrated its ability to recover the simulated parameters from the genealogical and phenotypic information. Computationally, the additional complexities beyond the standard mixed-model are the calculation of the partial inbreeding coefficients from the Mendelian decomposition of inbreeding and the calculation and storage of the ${\mathbf{K^{\prime}K}}$ block. First, the tabular method that was implemented for the inbreeding decomposition described in [12] involves the construction of a square matrix with the dimension of the pedigree. Thus, for computational feasibility, the partial inbreeding coefficients of each individual were calculated sequentially from a reduced pedigree that only included its ancestors. Second, the ${\mathbf{K^{\prime}K}}$ matrix is more dense than is the ${\mathbf{Z^{\prime}Z}}$ block and it requires more memory storage and computational time. Nevertheless, the contributions of the partial inbreeding coefficients to the ${\mathbf{K^{\prime}K}}$ matrix were squared and, thus, the very small partial inbreeding coefficients generated almost null values, which could be discarded.

The posterior distributions of the inbreeding load variances for weights at 210 days in the Pirenaica and Rubia Gallega breeds indicated high variability among them, which is consistent with the variability of inbreeding depression among sire families reported by Carolino and Gama [7]. The posterior mean estimates were much larger (29,966.8 and 28,222.4 in Pirenaica and Rubia Gallega breeds, respectively) than those of the other variance components in the model, which was confirmed by the broad range of the prediction of the inbreeding loads. However, it should be noted that the model provides estimates of the inbreeding loads that must be understood as the inbreeding depression achieved by a fully inbred (100%) descendent. Logically, the predictions are not realistic and have to be rescaled. Thus, the expected inbreeding depression from an ancestor that generates a partial inbreeding coefficient of 0.10 and has a predicted inbreeding load of − 100 kg is − 10 kg.

The analysis of the beef cattle datasets indicated that, for most of the individuals, the prediction of their inbreeding load was negative, which is consistent with several studies that have analyzed the inbreeding depression in body weights of beef cattle [7, 37–39]. However, a significant proportion of the individuals (10.52% in Pirenaica, and 22.54% in Rubia Gallega) had a positive prediction of their inbreeding loads. In spite of the prediction error variance of the inbreeding loads, these figures may indicate that a relevant proportion of the individuals might have had an incremental effect on the trait of interest. Therefore, the potential inbreeding caused by mating between the descendants might not need to be avoided or could even be favored, at least for the analyzed trait. It should be noted that with a similar posterior mean estimate of the variance component (29,966.8 and 28,222.4), the prediction of the inbreeding load effects was more variable in the Pirenaica (SD: 58.6 kg) than in the Rubia Gallega (SD: 41.7) breed. This was due to the different amounts of pedigree information available for the estimation of the inbreeding loads (5.9 vs. 3.9 generations in Pirenaica and Rubia Gallega breeds, respectively). A deeper pedigree generates more partial inbreeding coefficients (Table 1) and provides more reliable information for the prediction of the inbreeding loads, which is reflected in larger variances for the predictors.

The additive nature of the inbreeding loads implies that it can be genetically correlated with other traits such as the direct additive effect on the analyzed traits. The mean posterior estimate of the genetic correlation between the inbreeding loads and the direct additive effect in the Pirenaica breed was clearly negative (− 0.43) and the posterior probability that it was negative was higher than 0.99. In contrast, the estimate of the genetic correlation in the Rubia Gallega breed was near zero. To our knowledge, to date there are no published studies that provide estimates of the correlations between additive genetic and inbreeding load effects in livestock breeds, although Carolino and Gama [7] did not find a significant correlation between the breeding values of the sires and the inbreeding depression associated with them. The only study available which provided an estimate of the genetic correlation [40], although retracted, suggested a very small, negative genetic correlation [41]. A possible explanation of this negative correlation is that older individuals may have a smaller additive genetic effect due to selection and a higher inbreeding load as they are more exposed to purging than recent ancestors. This hypothesis may also explain the differences in the estimates of the genetic correlation between the Pirenaica and Rubia Gallega breeds, since the depth of the pedigree in the Pirenaica was larger, and, therefore, older individuals with more opportunities of purging were included. Regardless of its cause, if the negative correlation is confirmed, it will mean that individuals with high breeding values tend to cause worse inbreeding depression if their descendants are inbred.

Finally, it is worth noting that the assumed model uses a prior distribution of the inbreeding loads centered at zero. Further research is necessary to define alternative prior distributions that allow the mean of the inbreeding loads to differ from zero.

## Conclusions

Our study proposes a mixed-model approach that includes individual inbreeding depression loads, direct additive effects and the covariance between them. The approach was applied to simulated data and two datasets with records on weaning weight from beef cattle breeds (Pirenaica and Rubia Gallega) in Spain. The results demonstrated the ability of the model to recover the simulated parameters and to provide prediction of the individual inbreeding loads of candidates for selection without inbred progeny.

## Supplementary information

**Additional file 1: Figure S1.** Plot of the Gibbs Sampler chains for the inbreeding load variance in the Pirenaica Breed.

**Additional file 2: Figure S2.** Plot of the Gibbs Sampler chains for the inbreeding load variance in the Rubia Gallega Breed.

**Additional file 3: Figure S3.** Evolution of the number of inbred and non-inbred individuals and the average percentage of inbreeding from 1975 to 2017 in the Pirenaica Breed.

**Additional file 4: Figure S4.** Evolution of the number of inbred and non-inbred individuals and the average percentage of inbreeding from 1975 to 2017 in the Rubia Gallega Breed.

## Data Availability

The datasets and software used in the study are available from the corresponding author on reasonable request.
