# Supplementary figures and images for: A multivariate analysis with direct additive and inbreeding depression load effects
Source: Genet Sel Evol. 2019 Dec 26;51:78. doi: 10.1186/s12711-019-0521-3 (PMC6933709; doi:10.1186/s12711-019-0521-3)

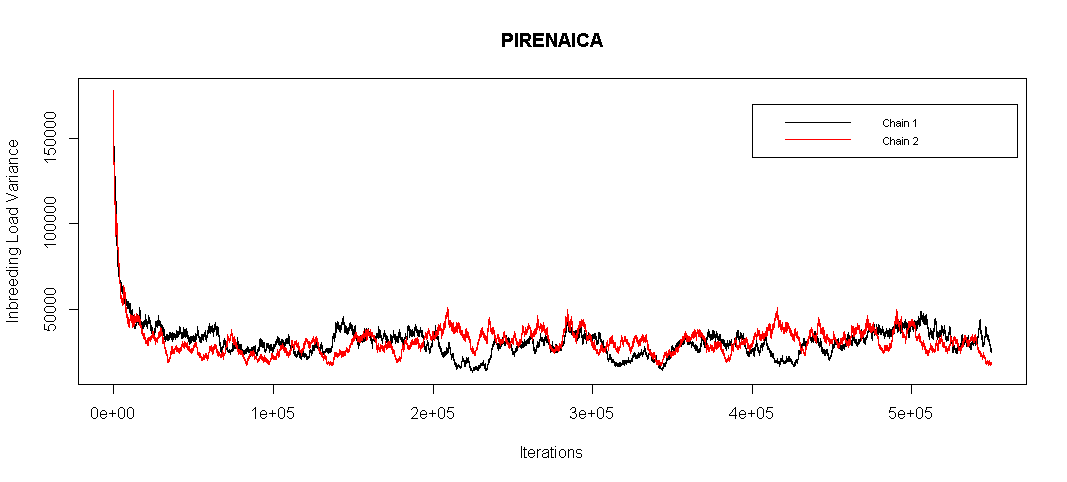

Supplement: Supplementary file 1 — Additional file 1: Figure S1. Plot of the Gibbs Sampler chains for the inbreeding load variance in the Pirenaica Breed. [file 12711_2019_521_MOESM1_ESM.docx]

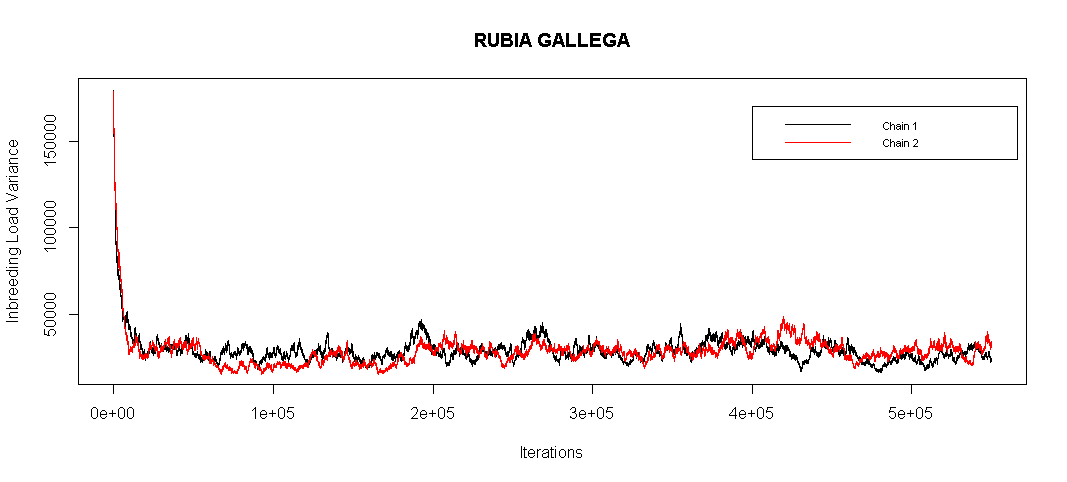

Supplement: Supplementary file 2 — Additional file 2: Figure S2. Plot of the Gibbs Sampler chains for the inbreeding load variance in the Rubia Gallega Breed. [file 12711_2019_521_MOESM2_ESM.docx]

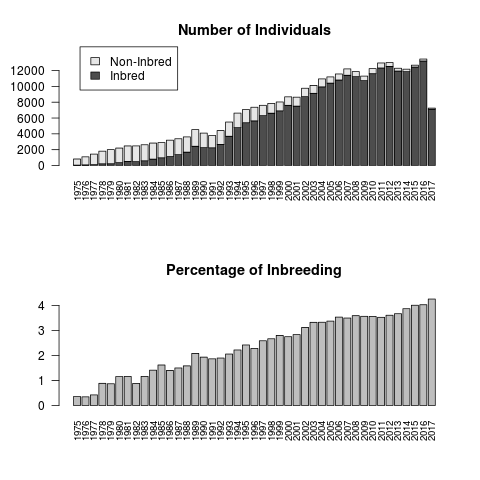

Supplement: Supplementary file 3 — Additional file 3: Figure S3. Evolution of the number of inbred and non-inbred individuals and the average percentage of inbreeding from 1975 to 2017 in the Pirenaica Breed. [file 12711_2019_521_MOESM3_ESM.docx]

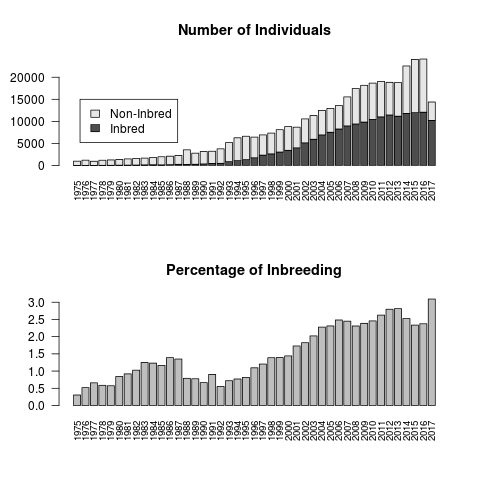

Supplement: Supplementary file 4 — Additional file 4: Figure S4. Evolution of the number of inbred and non-inbred individuals and the average percentage of inbreeding from 1975 to 2017 in the Rubia Gallega Breed. [file 12711_2019_521_MOESM4_ESM.docx]
